# Plg-R_KT_ Expression in Human Breast Cancer Tissues

**DOI:** 10.3390/biom12040503

**Published:** 2022-03-26

**Authors:** Lindsey A. Miles, Stan Krajewski, Nagyung Baik, Robert J. Parmer, Barbara M. Mueller

**Affiliations:** 1 Department of Molecular Medicine, Scripps Research Institute, La Jolla, CA 92037, USA; lmiles@scripps.edu (L.A.M.); nagyung@scripps.edu (N.B.); 2 Cellstan-Immunoquant Inc., Oceanside, CA 92054, USA; stankrajewski@cellstan.com; 3 Department of Medicine, Veterans Administration San Diego Healthcare System, University of California San Diego, San Diego, CA 92161, USA; rparmer@ucsd.edu; 4 San Diego Biomedical Research Institute, San Diego, CA 92121, USA

**Keywords:** breast cancer, plasminogen, Plg-R_KT_, tissue microarrays, tumor microenvironment

## Abstract

The plasminogen activation system regulates the activity of the serine protease, plasmin. The role of plasminogen receptors in cancer progression is being increasingly appreciated as key players in modulation of the tumor microenvironment. The interaction of plasminogen with cells to promote plasminogen activation requires the presence of proteins exposing C-terminal lysines on the cell surface. Plg-R_KT_ is a structurally unique plasminogen receptor because it is an integral membrane protein that is synthesized with and binds plasminogen via a C-terminal lysine exposed on the cell surface. Here, we have investigated the expression of Plg-R_KT_ in human breast tumors and human breast cancer cell lines. Breast cancer progression tissue microarrays were probed with anti-Plg-R_KT_ mAB and we found that Plg-R_KT_ is widely expressed in human breast tumors, that its expression is increased in tumors that have spread to draining lymph nodes and distant organs, and that Plg-R_KT_ expression is most pronounced in hormone receptor (HR)-positive tumors. Plg-R_KT_ was detected by Western blotting in human breast cancer cell lines. By flow cytometry, Plg-R_KT_ cell surface expression was highest on the most aggressive tumor cell line. Future studies are warranted to address the functions of Plg-R_KT_ in breast cancer.

## 1. Introduction

The plasminogen activation system regulates the activity of the serine protease, plasmin. Plasminogen activation to plasmin is carried out by plasminogen activators, notably the urokinase-type plasminogen activator (uPA). The plasminogen activation system is critical for the dissolution of fibrin clots, plays a major role in processes of tissue remodeling and cell migration [1,2,3], and has been compellingly implicated in the biology of breast cancer [4,5]. Indeed, several components of this system including the protease uPA, its receptor, uPAR, and the plasminogen activator inhibitor-1 (PAI-1) are strong, independent markers of a poor prognosis in breast cancer [6,7,8,9].

Efficient plasminogen activation requires coordinated activity on the cell surface [10,11]. The interaction of plasminogen with cells to promote plasminogen activation requires the presence of proteins exposing C-terminal lysines on the cell surface [12]. The role of plasminogen receptors in cancer progression is being increasingly appreciated (reviewed in [13,14,15,16,17,18]).

Previously, using a proteomics-based approach, we discovered the plasminogen receptor, Plg-R_KT._ Among cell surface plasminogen binding proteins Plg-R_KT_ is structurally unique in that it is an integral membrane protein that is synthesized with and binds plasminogen via a C-terminal lysine exposed on the cell surface [19,20]. Plg-R_KT_ is highly colocalized with uPAR [19,21] and promotes uPA-dependent plasminogen activation [22]. Furthermore, Plg-R_KT_ regulates fibrinolysis, pro-MMP cleavage [22], and leukocyte recruitment [22,23,24,25], all of which may play a role in breast cancer progression. Here, we have investigated the expression of Plg-R_KT_ in human breast tumors. Our results suggest that Plg-R_KT_ is expressed on human breast tumors and expression increases with tumor progression.

## 2. Materials and Methods

### 2.1. Tissue Microarrays

We used the human breast (cancer) tissue microarray IMH 364 from Novus, Centennial, CO, USA. We also used the National Cancer Institute (NCI) Cancer Diagnosis Program (CDP) 2nd Generation Breast Cancer Progression (BCP) Tissue Microarrays (CDP-BCP-TMA) case sets 4, 6, and 8, from University of Virginia, CHTN (Cooperative Human Tissue Network), Charlottesville, VA 22908, USA, http://www.chtn.org (Accessed on 20 February 2022).

### 2.2. Antibodies

Anti-Plg-R_KT_ (mAb 7H1) is a pan-specific antibody that we raised in mice against a synthetic peptide corresponding to the 9 C-terminal amino acids of rat Plg-R_KT_ [22]. Anti-β actin antibody was from LI-COR (Lincoln, NE, USA). Normal mouse IgG2_a_ was from Southern Biotech (Birmingham, AL, USA).

### 2.3. Immunohistochemistry and Calculation of Immunoscores

Immunohistochemistry was performed as described [26]. Briefly, immunostaining of TMAs was carried out using the Anti-Mouse Envision-Plus-HRP system (Dako, Troy, MI, USA) with a Dako Universal Staining System automated immunostainer. In controls, the immunostaining procedure was performed in parallel by absorbing the antibody with the synthetic peptide used for immunization. To quantify Plg-R_KT_ expression we calculated anti-Plg-R_KT_ immunoscores as described [27]. 

### 2.4. Cell Lines

The MCF-7 hormone receptor positive human cancer cell line was obtained from the America Type Culture Collection (ATCC, Manassas, VA, USA) and cultured in DMEM containing 10% fetal bovine serum (FBS), 1% L-Glutamine, 1% sodium pyruvate, 1% nonessential amino acids (NEAA) and 2% vitamins. The MCF-10A normal human breast cell line was from the University of Colorado and cells were cultured in DMEM:F12 containing 5% horse serum, 1% L-Glutamine, 20 ng/mL EGF, 0.5 µg/mL hydrocortisone, 100 ng/mL cholera toxin and 10 µg/mL insulin. The triple negative SUM159PT human breast cancer cell line was from BioIVT (Westbury, NY, USA) and was cultured in Ham’s F-12 media containing 5% FBS, 10 mM HEPES, 1 µg/mL hydrocortisone and 5 µg/mL insulin. The MDA-MB-231 triple negative human breast cancer cell line was obtained from ATCC and was cultured in Leibovitz’s L-15 media containing 10% FBS and 10 mM HEPES without CO_2_. The MDA-MB-231 mfp line was obtained by establishing orthotopic xenograft tumors of the invasive human breast cancer line, MDA-MB-231, in the mammary fat pad of SCID mice. Tumor cells were recovered and cultured to provide a propagatable subpopulation, referred to as 231mfp cells [28].

### 2.5. Western Blotting

Cells were lysed in radio-immunoprecipitation assay (RIPA) buffer containing an anti-protease and anti-phosphate cocktail (Thermo Fisher Scientific, Waltham, MA, USA) and electrophoresed on 4–12% polyacrylamide gels under reducing conditions followed by electrotransfer to nitrocellulose membranes. The membranes were incubated with primary antibodies, then washed with phosphate buffered saline (PBS) containing 0.1% Tween-20 and then incubated with species specific IRDye^®^ 680RD/800CW-conjugated second antibodies. Immunoreactive bands were visualized with the Odyssey Imaging System (LI-COR). Densitometric analysis was performed using the software Image Studio™ Lite Software 5.2 (LI-COR).

### 2.6. Flow Cytometric Analysis

Cell lines were stained with either anti-Plg-R_KT_ mAB, normal mouse IgG2_a_ or unstained. The anti-Plg-R_KT_ and isotype control antibody were directly labeled with PE-Cy7 Conjugation Kit from Abcam (Boston, MA, USA). The stained cells were acquired in a NovoCyte (ACEA Biosciences, San Diego, CA, USA) and analyzed with FlowJo software (Tree Star Inc., Ashland, OR, USA). Viable cells (propidium iodide and annexin V negative) were gated from nonviable cells.

Quantitative flow cytometric equilibrium binding of fluorescein isothiocyanate (FITC)-plasminogen to cells was analyzed using beads impregnated with FITC as described [19]. Nonspecific (FITC)-plasminogen binding in the presence of epsilon amino caproic acid (EACA) was subtracted from total binding to obtain specific binding for Scatchard analysis.

### 2.7. Statistical Analysis

Data are as mean ± SEM. Significance was determined by ANOVA with Tukey’s multiple comparison test. Statistical calculations were performed using the Prism 5.0 software program (GraphPad Software, San Diego, CA, USA). 

## 3. Results

### 3.1. Plg-R_KT_ Expression in Human Breast Cancer Tissue Microarrays

**Figure 1:**
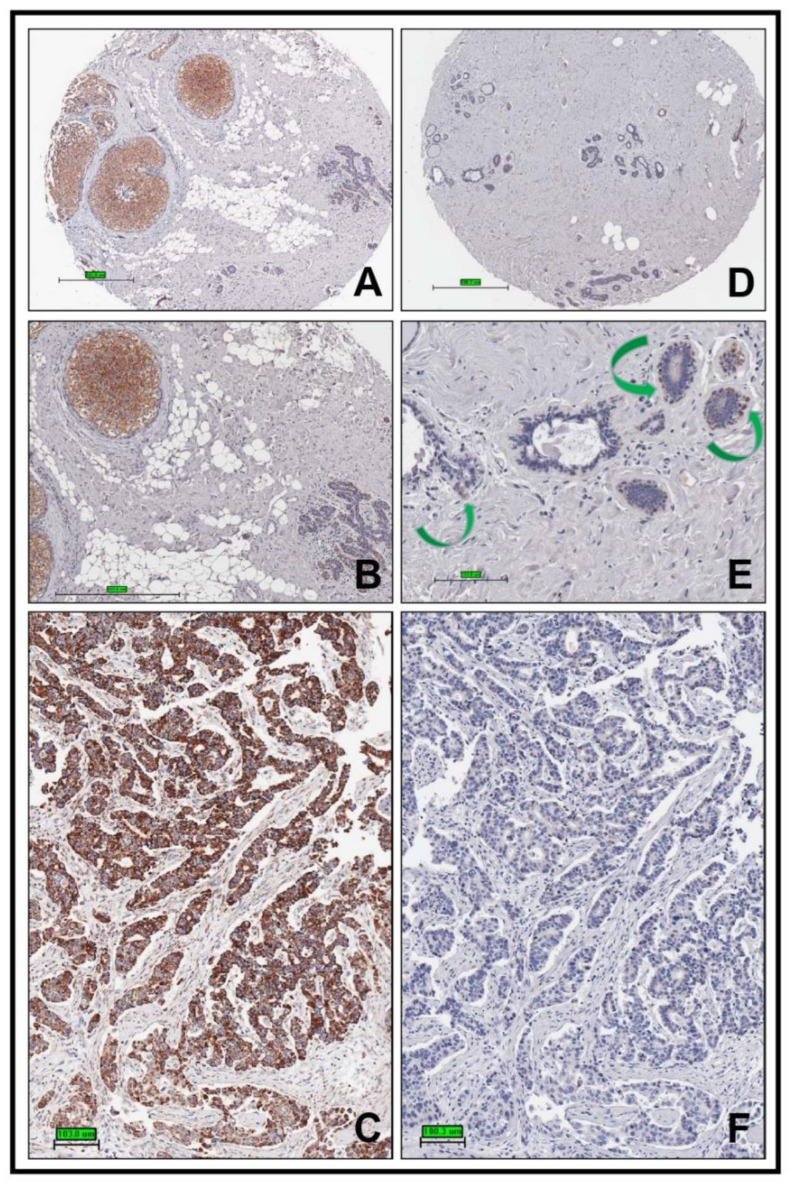
Plg-R_KT_ is highly expressed in human ductal carcinoma in situ and invasive ductal carcinoma. A core from a patient with ductal carcinoma in situ was stained with anti-Plg-R_KT_ mAb using paraffin immunocytochemistry (**A**) and enlarged in (**B**). Cores from the same patient showing invasive ductal carcinoma (**C**) and from a 60-year-old healthy female control subject ((**D**) and enlarged in panel (**E**)) were stained in the same way. Green arrows in panel E indicate cells with macrophage morphology that also stain with anti-Plg-R_KT_ mAb. A specificity control in which the core shown in Panel C was stained with anti-Plg-R_KT_ mAb that had been absorbed with the Plg-R_KT_ peptide used for immunization is shown in Panel F. Original magnifications, ×100 (**A**,**D**), ×200 (**B**) to ×400 (**C**,**E**,**F**).

To determine Plg-R_KT_ expression in human breast cancer, we initially probed the human breast tissue (cancer) tissue microarray IMH 364 from Novus. We found faint to moderate expression of Plg-R_KT_ in human ductal carcinoma in situ (Figure 1A,B) and highest expression in invasive ductal carcinoma (Figure 1C) compared to normal adjacent tissue. Weak expression of Plg-R_KT_ was detected in normal breast ducts and lobules from a healthy control subject (Figure 1D,E). No staining was detectable after preabsorbing the antibody with the Plg-R_KT_ peptide used for immunization (Figure 1F). In addition, in normal noncancerous breast tissue, periacinar cells exhibiting macrophage morphology also stained with anti Plg-R_KT_ antibody (Figure 1E, arrows).

**Figure 2:**
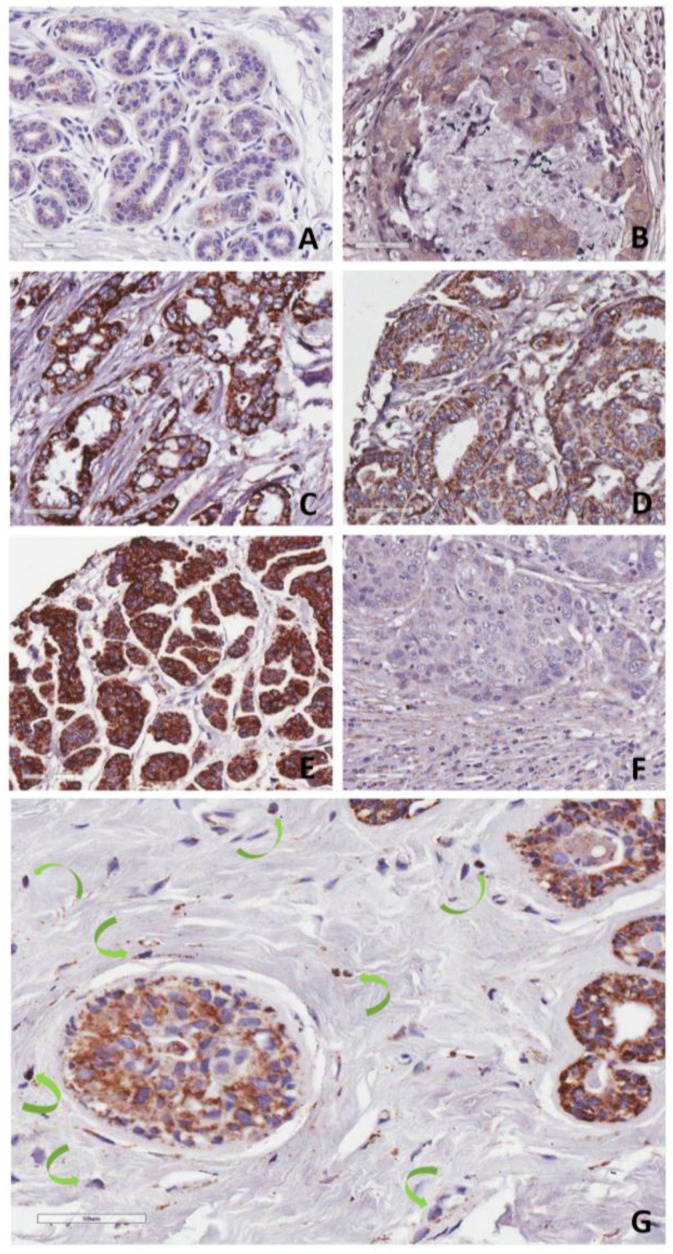
Plg-R_KT_ expression in human mammary tissues. (**A**) Ductal epithelial cells in normal breast with light granular Plg-R_KT_ staining, (**B**) ductal carcinoma in situ with faint to moderate staining, (**C**) invasive, LN-positive HR-positive breast tumor, (**D**) invasive, HR-positive tumors with distant metastases show moderate and (**E**) strong granular Plg-R_KT_ staining. (**F**) Invasive, LN-positive triple-negative breast tumor exhibits very fine granular Plg-R_KT_ staining of tumor cells and faint to moderate staining of the reactive stroma. (**G**) Ductal carcinoma in situ showing staining of TAMs (identified by morphology and indicated with green arrows). Plg-R_KT_ by IHC using anti-Plg-R_KT_ monoclonal antibody 7H1. Shown are representative tissues from the CDP breast cancer progression TMA. Original magnifications, ×100 (**A**,**E**), ×200 (**C**,**D**,**F**) to ×400 (**B**,**G**).

To further explore Plg-R_KT_ expression during progression of human breast cancer we used the NCI Cancer Diagnosis Program Breast Cancer Progression Tissue Microarray (BCP-TMA), which has tissues and associated pathological and clinical outcome data from the Cooperative Breast Cancer Tissue Resource. Three nonoverlapping case sets of the BCP-TMA were probed using immunohistochemistry with anti-Plg-R_KT_ mAb. Staining of representative tissues is shown in Figure 2. Plg-R_KT_ was weakly expressed in ductal epithelial cells in normal breast tissue with light granular Plg-R_KT_ staining (Figure 2A). Plg-R_KT_ expression increased with anatomical stage of breast tumors. Ductal carcinoma in situ showed faint to moderate staining (Figure 2B). Invasive, lymph node (LN)-positive hormone receptor (HR)-positive breast tumor (Figure 2C) and invasive, HR-positive tumors with distant metastasis showed moderate (Figure 2D) to strong granular Plg-R_KT_ staining (Figure 2E). In contrast, an invasive, LN-positive triple-negative (lacking estrogen receptor (ER), progesterone receptor (PR) and human EGF receptor 2 (HER2)) breast tumor showed very light granular Plg-R_KT_ staining of tumor cells and light to moderate staining of the reactive stroma (Figure 2F). In addition, strong Plg-R_KT_ staining was observed on structures with morphologies consistent with tumor associated macrophages (TAMs) (Figure 2G).

**Figure 3:**
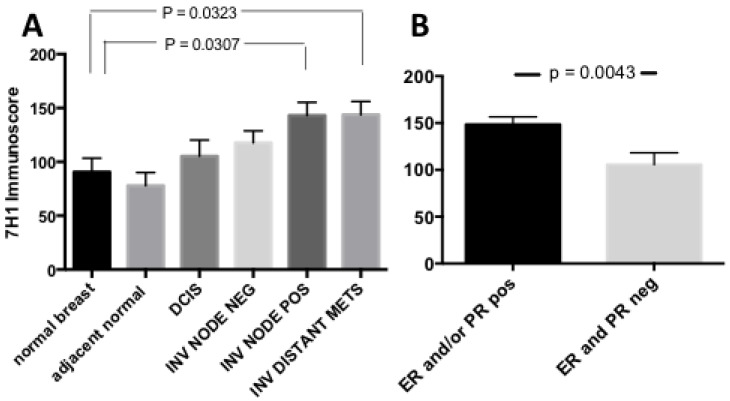
Plg-R_KT_ in human breast cancer progression. Anti-Plg-R_KT_ mAB immunoscores for tissues presented in the CDP breast cancer progression TMA. (**A**) By anatomical stage: normal breast from nonbreast cancer (n = 23), normal adjacent breast from breast cancer patient (n = 12), DCIS (n = 22), invasive tumor without lymph node involvement (n = 48), invasive tumor with lymph node involvement (n = 50) and invasive tumor with distant metastasis (n = 45). (**B**) By hormone receptor status: ER and/or PR-positive (n = 95), ER and PR-negative (n = 43). Values are means and SEM, significance by ANOVA with Tukey’s multiple comparison test.

To quantify Plg-R_KT_ expression we calculated anti-Plg-R_KT_ immunoscores [27]. Briefly the number of Plg-R_KT_ positive cells and the total number of cells were counted in 10 random high-power fields. The intensity of immunostaining was scored as 0 = negative; 1+ = weak; 2+ = moderate; and 3+ = strong. Immunoscores were calculated as the percentage of positive cells (0 to 100) multiplied by staining intensity score (0/1/2/3) to yield scores ranging from 0 to 300. Plg-R_KT_ immunoscores increased with anatomical stage (Figure 3A) and were highest in invasive tumors with lymph node (LN) involvement and/or distant metastasis. Plg-R_KT_ expression was also compared with expression of the biomarkers ER, PR, and HER2, as annotated in the BCP-TMA. For analysis, ER and/or PR-positive tumors were treated as one group, (hormone receptor (HR) positive tumors). We found that Plg-R_KT_ expression was significantly higher in HR-positive tumors than in HR-negative tumors (Figure 3B). Immunoscores for Plg-R_KT_ in ER/PR/HER2-negative (triple negative) tumors were low and were not significantly different from HR-negative, HER2 positive tumors (Supplementary Figure S1). Plg-R_KT_ expression in HER2-positive tumors was moderate to high (Supplementary Figure S1), however the majority of Plg-R_KT_ high scoring HER2 tumors were also HR-positive (e.g., Supplementary Figure S1). It was also noteworthy that only four tumors were negative for Plg-R_KT_ expression and these were also HR-negative. Together, these results demonstrate that Plg-R_KT_ is widely expressed in human breast tumors, that its expression is increased in tumors that have spread to draining lymph nodes and distant organs, and that Plg-R_KT_ expression is most pronounced in HR-positive tumors. 

### 3.2. Plg-R_KT_ Expression in Human Breast Cancer Cell Lines

**Figure 4:**
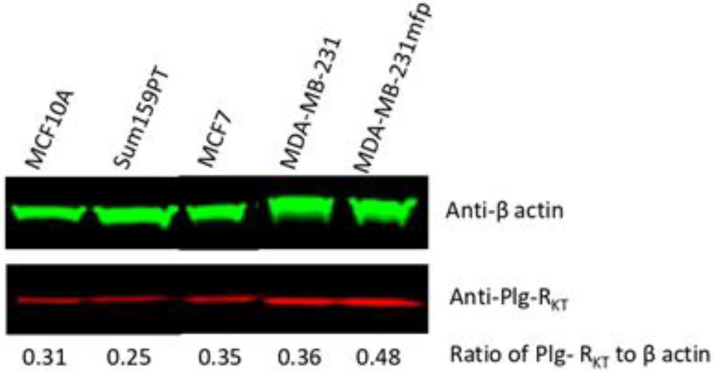
Western blot analysis of expression of Plg-R_KT_ in human breast cancer cell lines. The cells were lysed in RIPA buffer and 30 µg were electrophoresed on 4–12% gradient gels under reducing conditions and Western blotted with anti-Plg-R_KT_ mAB and anti-actin.

We assessed Plg-R_KT_ expression on a number of established human cell lines. Using Western blotting we found the receptor to be expressed in all cell lines tested, including the HR-positive normal breast cell line MCF10A and the tumorigenic HR-positive MCF7 line as well as tumorigenic triple negative breast cancer cell lines, SUM159PT and MDA-MB-231 (Figure 4). These data show that the receptor is expressed on human breast cancer cell lines including triple-negative cell lines which are examples of highly aggressive tumors. By flow cytometry, positive signals were obtained for all cell lines tested (data not shown).

**Figure 5:**
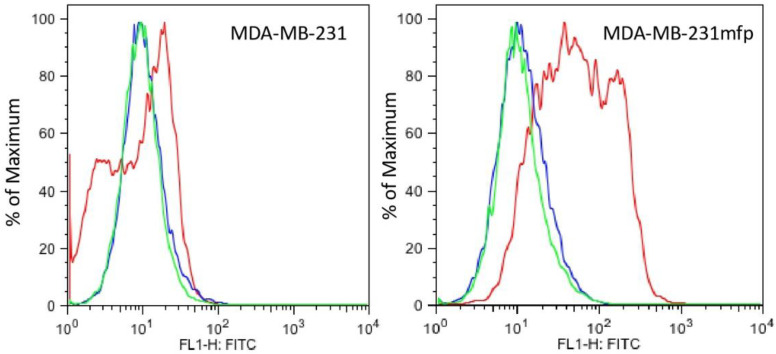
Plg-R_KT_ expression is enhanced on MDA-MB-231mfp cells. MDA-MB-231mfp cells and MDA-MB-231 cells were analyzed, using FACS analysis, for staining with anti-Plg-R_KT_ mAb7H1 (red) compared to isotype control (blue) or unstained (green).

We have previously examined the impact of the microenvironment on breast cancer by establishing orthotopic xenograft tumors of the invasive human breast cancer line, MDA-MB-231, in the mammary fat pad (mfp) of SCID mice. Tumor cells were recovered and cultured to provide a propagatable subpopulation, here referred to as MDA-MB-231mfp cells [28,29]. A total of 231mfp cells exhibited a dramatic increase in tumor growth in vivo. The 231mfp tumors also produced significantly more lung and lymph node metastases than tumors from the parental cell line, MDA-MB-231 [28,29]. In the MDA-MB-231 model we found that the highly metastatic subline MDA-MD-231mfp expressed more Plg-R_KT_ than the parental MDA-MB-231 line (Figure 4 and Figure 5). The MDA-MB-231 mfp cell line also exhibited markedly enhanced plasminogen binding ability compared to the parental MDA-MB-231 cells. In FACS analysis specific plasminogen binding to MDA-MB-231mfp cells was increased 3-fold, compared to MDA-MB-231 cells (Supplementary Figure S2). Quantitative FACS analysis yielded a B_max_ of 3.8 ± 0.5 × 10^5^ plasminogen binding sites with a Kd of 1.2 μM for the MDA-MB-231mfp cells and a B_max_ of 1.2 ± 0.3 × 10^5^ sites with a Kd of 1.5 μM for the parental cells. Comparing the FACS data for plasminogen binding and anti-Plg-R_KT_ mAB binding, we reason that most of the increased plasminogen binding on the MDA-MB-231mfp cells is to Plg-R_KT_. However, other plasminogen receptors/binding proteins have been described [16,17] and some plasminogen binding to the surfaces of the cells may be due to other receptors. This will be addressed in future studies. Together, these data show that the receptor is expressed on human breast cancer cell lines and receptor expression appears to increase with tumor progression.

## 4. Discussion

The interaction of plasminogen with cancer cells plays a key role in progression of breast cancer and other cancers [13,14,15,16,17,18]. Here, we provide the first report that the plasminogen receptor, Plg-R_KT_, is widely expressed in human breast tumors, that its expression is increased in tumors that have spread to draining lymph nodes and distant organs, and that Plg-R_KT_ expression is most pronounced in HR-positive tumors. In addition, we provide the first evidence that Plg-R_KT_ is present on the surface of breast cancer cell lines.

Triple negative breast cancer TMA samples had low Plg-R_KT_ expression whereas triple negative human breast cancer cell lines (MDA-MB-231 and MDA-MB-231mfp) expressed higher levels of Plg-R_KT_ than the HR-positive MCF7 cell line. It is important to note that the TMA was constructed using primary breast tumor tissues from women that were diagnosed and underwent breast cancer surgery. Tissues from advanced tumors including tumors from metastatic sites were not represented in the tumor microarray (because these tumors are not subjected to surgery and such tissues are rarely available). A likely reason for the discrepancy in Plg-R_KT_ expression in the TMA and established cancer cell lines is that the MDA-MB-231 cell line was established from a metastatic tumor (pleural effusion) and cell lines can also become more aggressive following multiple passages.

Our results suggest that Plg-R_KT_ could provide a new marker for staging of human breast tumors. Notably, a recent report identified over expression of Plg-R_KT_ as a novel marker in inflammatory breast cancer [30]. Interestingly, Plg-R_KT_ mRNA expression is increased in gliomas of higher grades [17]. Future studies are warranted to investigate the potential of Plg-R_KT_ as a marker in other cancers. 

In the MDA-MB-231 model we found that the highly metastatic subline MDA-MB-231mfp expressed more Plg-R_KT_ than the parental MDA-MB-231 line, suggesting that Plg-R_KT_ may promote breast cancer progression. Furthermore, the MDA-MB-231mfp cell line exhibited markedly enhanced plasminogen binding ability compared to the parental MDA-MB-231 cells. In FACS analysis specific plasminogen binding to 231mfp was increased 3-fold, compared to MDA-MB-231 cells (Supplementary Figure S2). This increase in plasminogen binding is very significant because each plasminogen binding site stimulates plasminogen activation 11-60-fold [10] and the Kd for the interaction indicates that these binding sites will be substantially occupied at the plasminogen concentration in the interstitial fluid in the tumor microenvironment (2 μM [31]). 

Mechanistically, Plg-R_KT_ may regulate breast cancer progression by binding plasminogen on cancer cell surfaces to promote its activation to plasmin, leading to fibrinolysis, extracellular matrix degradation and activation of proenzymes of MMPs. We also found Plg-R_KT_ expression on tumor associated macrophages (TAMs). Plg-R_KT_ regulates macrophage recruitment [22,23,24] and also promotes plasminogen-dependent polarization to the M2 like phenotype and macrophage intracellular signaling [25]. Thus, regulation of TAM function [32] is an additional mechanism by which Plg-R_KT_ may regulate tumor progression.

Plg-R_KT_ may also have protective functions in breast cancer independent of its plasminogen binding capacity. Plg-R_KT_ deletion in mice results in complete inability to lactate, due to decreased proliferation of epithelial cells [33]. A preponderance of evidence from epidemiological studies points to an inverse relationship between the length of the breastfeeding period and breast cancer risk, suggesting that breastfeeding protects mothers from development of breast cancer (reviewed in [34,35,36,37]). One proposed mechanism is that differentiation of epithelial cells to generate milk diminishes the vulnerability of breast tissue toward the carcinogenic effects of estrogens [38]. Thus, Plg-R_KT_-dependent lactational development may prevent the development of breast cancer. Future studies are warranted to address the functions of Plg-R_KT_ in breast cancer.

## Data Availability

Please contact Stan Krajewski at stankrajewski@cellstan.com or Barbara Mueller at bmueller.sandiego@gmail.com for IHC data.

## Supplementary Materials

The following supporting information can be downloaded at: https://www.mdpi.com/article/10.3390/biom12040503/s1, Figure S1: Plg-R_KT_ in human breast cancer progression, Figure S2, Plasminogen binding is enhanced on MDA-MB-231mfp cells.
