# Supplementary material for: Plg-RKT Expression in Human Breast Cancer Tissues
Source: Biomolecules. 2022 Mar 26;12(4):503. doi: 10.3390/biom12040503 (PMC9028288; doi:10.3390/biom12040503)
Supplement: Supplementary file 1 [file biomolecules-12-00503-s001.zip › biomolecules-1635195-supplementary.pdf]

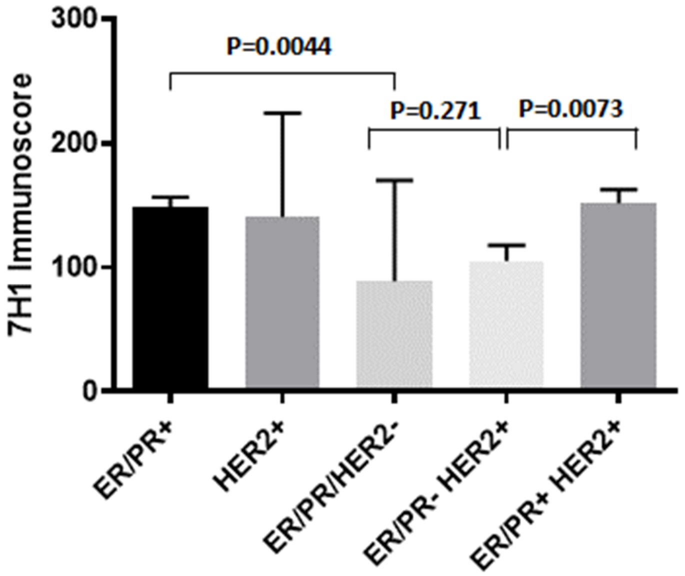

Supplementary Figure S1. Plg-R_{KT} in human breast cancer progression. Anti-Plg-R_{KT} mAB immunoscores for tissues presented in the CDP breast cancer progression TMA. By hormone receptor status: ER and/or PR positive (n=95), HER2 positive (n= 79), ER,PR and HER2-negative (n=18), ER/PR negative, Her2 positive (n=25), ER, PR and HER2 positive (n=54). Values are means and SEM, significance by ANOVA with Tukey's multiple comparison test.

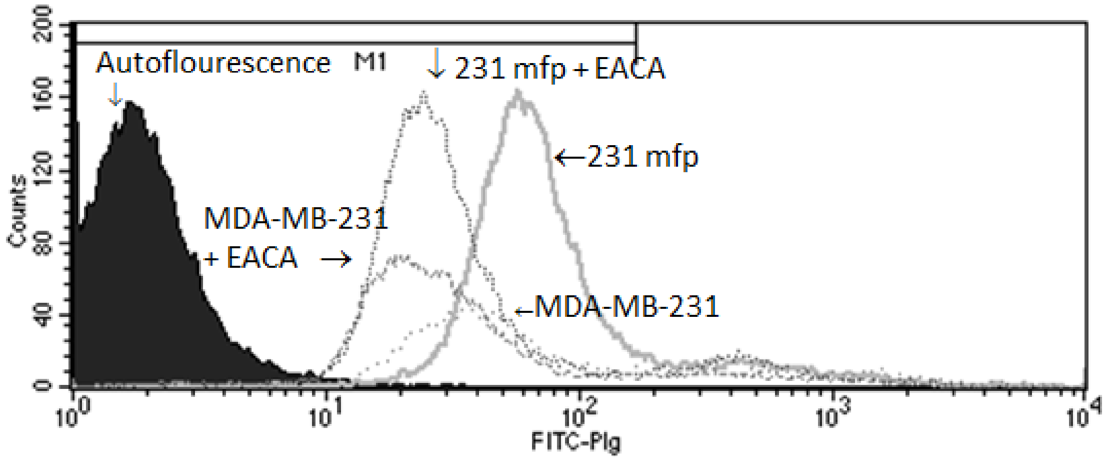

Supplementary Figure S2. Plasminogen binding is enhanced on MDA-MB-231mfp cells. MDA-MB-231 and MDA-MB-231mfp cells were analyzed in FACS analysis with 0.5 μM FITC-plasminogen in the presence or absence of 0.2 M epsilon aminocaproic acid (EACA) to assess non-specific binding. Viable cells were gated from nonviable cells. Histogram plots of FITC-plasminogen binding and autofluorescence are shown. Specific plasminogen binding was blocked in the presence of EACA.
